# Comparative Transcriptome Analysis of the Pest *Galeruca daurica* (Coleoptera: Chrysomelidae) Larvae in Response to Six Main Metabolites from *Allium mongolicum* (Liliaceae)

**DOI:** 10.3390/insects15110847

**Published:** 2024-10-29

**Authors:** Ling Li, Jinwei Li, Haichao Wang, Yanyan Li, Ruiwen Dong, Baoping Pang

**Affiliations:** 1 Research Center for Grassland Entomology, Inner Mongolia Agricultural University, Hohhot 010020, China; lijinwei611@163.com (J.L.); wanghc@imau.edu.cn (H.W.); liyanyan@imau.edu.cn (Y.L.); pangbp@imau.edu.cn (B.P.); 2 Siziwang Grassland Station in Wulanchabu City, Ulanqab 011800, China; szwqcyz@163.com

**Keywords:** *Galeruca daurica*, transcriptome sequencing, differentially expressed genes, host adaptability, metabolites

## Abstract

Plants are important ecological factors and food resources, which can significantly affect the occurrence and distribution of insects. The metabolites in host plants can affect the feeding, spawning, and avoidance behaviors of herbivorous insects. *Galeruca daurica* (Joannis) is a phytophagous pest that has seriously occurred in the desert steppe of Inner Mongolia in recent years, only infesting the leaves of *Allium* plants. In order to clarify the effects of plant metabolites on the gene expression in *G. daurica* larvae at the transcriptome level, we fed the larvae of *G. daurica* with *Allium tuberosum* leaves soaked in 10% DMSO solutions containing d-galactose, β-d-glucopyranose, l-rhamnose, isoquercitrin, isoflavone, and rutin, respectively, used the larvae fed on *A. tuberosum* leaves soaked in a 10% DMSO solution as the control, and screened out the differentially expressed genes (DEGs) by performing high-throughput transcriptome sequencing. The results showed that a total of 291 DEGs were identified compared to the solvent control (DMSO), including 130, 34, 29, 21, 72, and 97 in the isoquercitrin, isoflavone, rutin, d-galactose, β-d-glucopyranose, and l-rhamnose treatment groups, respectively. GO and KEGG enrichment analysis showed that most DEGs were enriched in various metabolic pathways, implying that these six main primary and secondary metabolites in *Allium* plants may affect various metabolic processes in the larvae of *G. daurica*.

## Simple Summary

*Galeruca daurica* (Coleoptera: Chrysomelidae) has become one of the most important insect pests in the grasslands of Inner Mongolia, China. Herbivorous insects have a very close relationship with host plants, which constitute the food source, mating site, oviposition site, and habitat of all or part of their life cycle, and the digestion and utilization of nutrients and the adaptation of secondary metabolites are the key factors for insects to establish populations. In this study, after the larvae of *G*. *daurica* fed, respectively, on foods containing six different plant metabolites (three primary metabolites and three secondary metabolites), a total of 291 differentially expressed genes (DEGs) were identified compared to the solvent control, including 130, 34, 29, 21, 72, and 97 in the isoquercitrin, isoflavone, rutin, d-galactose, β-d-glucopyranose, and l-rhamnose treatment groups, respectively. GO and KEGG enrichment analysis showed that most DEGs were enriched in various metabolic pathways, implying that these six main primary and secondary metabolites in *Allium* plants may play crucial roles in various metabolic processes in the larvae of *G. daurica*.

## 1. Introduction

In the long-term evolutionary process, there is a close and complex relationship between herbivorous insects and host plants. On the one hand, host plants provide a source of nutrients for herbivorous insects, and on the other hand, they also affect the biological characteristics of herbivorous insects, such as growth and reproduction [1,2]. Many factors affect insect feeding, among which phytochemical properties are the main ones, including plant nutrients or primary metabolites and secondary metabolites [3,4]. Primary metabolites include carbohydrates, lipids, nucleic acids, and proteins. For example, feeding on carbohydrates, such as sucrose, glucose, and fructose, is beneficial for insect survival and reproduction [5,6]. Plant secondary metabolites are usually divided into three broad classes: terpenoids, phenolics (polyphenols), and alkaloids, among which phenolics are the largest and most diverse and most widely distributed class, which mainly include flavonoids, phenolic acids, stilbenes, and lignans [7]. Flavonoids, which also include flavones, flavanols, flavans, anthocyanins, and isoflavones, are secondary biomasses with predominantly anti-insect functions and have been shown to play an important role in the anti-insect process of plants [8,9]. For example, both flavonoids and isoflavonoids could protect plants against herbivorous insects by negatively affecting their behavior, growth, and development [10,11]. Flavonoids affected the nutrient accumulation in *Piezodorus guildinii* nymphs, making them unable to complete the molting process and causing death [12]. The addition of coumarin and cinnamic acid to the diet of *Helicoverpa armigera* larvae and *Spodoptera litura* larvae significantly increased the mortality of the tested insects [13]. In addition, flavonoids can inhibit digestion in insects as feeding deterrents [14]. Four flavonoids, isoglabratephrin, (+)-glabratephrin, tephroapollin-F, and lanceolatin-A, reduced the relative growth rate, relative consumption rate, and efficiency of conversion of ingested food by three stored grain insects [15].

*Allium* plants possess a variety of nutritional, biological, and health-promoting properties, including antimicrobial, antioxidant, anti-tumor, immunoregulatory, antidiabetic, and anti-inflammatory effects [16]. Some studies have indicated that a total of 23 flavonoids are identified from *Allium mongolicum*, among which the content of rutin and isoquercitrin was relatively large [17,18,19]. The sugar residues in *Allium* spp. are mainly composed of linear or branched glucose, rhamnose, galactose, xylose, and arabinose units [20]. Galactose is a significant nutrient for insect herbivores [21]. Rutin is a flavonoid found predominantly in different plant parts and exists as a glycoside (3-O-rhamnoglucoside/rutinose) of quercetin [22]. Rutin is shown to possess antioxidant activity [23]. Rutin and quercetin-3-glucoside suppressed the development and increased the mortality of *Lymantria dispar* [14]. Rutin prolonged the developmental duration of *Spodoptera frugiperda* [24] and *S. litura* larvae [25]. Quercetin increased the development time, pre-reproductive period, and mortality and reduced fecundity in *Acyrthosiphon pisum* [26], and it also negatively affected larval growth and pupal weight in *S. litura* [27]. However, quercetin was not yet reported as a phagostimulant [28].

Herbivorous insects can tolerate these toxic secondary substances because they have evolved various physiological mechanisms to avoid their harmful effects. Most insect herbivores depend heavily on enzymatic degradation for the neutralization of ingested plant secondary metabolites. Main detoxifying enzymes include cytochrome P450 (CYP450), glutathione S-transferase (GST), and carboxylesterase (CarE), among which CYP450 enzymes are the most extensively studied enzymes that effectively metabolize a wide variety of toxic substances [29,30]. Insect CYP450 genes can be induced by a variety of plant secondary metabolites [31]. In *Depressaria pastinacella*, CYP450 activity rose in response to the presence of furanocoumarins in its diet [32]. After injecting a tea saponin solution into the abdomen of adult *Locusta migratoria*, the activity of CYP450, GST, and CarE significantly increased [33]. Nevertheless, the molecular mechanism of herbivorous insects’ detoxification or tolerance to secondary plant compounds remains unclear.

**Figure 1:**
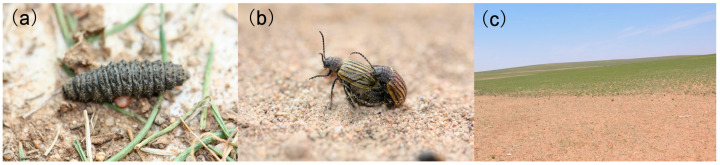
Pictures of *G. daurica* morphology and damage. (**a**) larvae; (**b**) adults; (**c**) damaged grassland.

*Galeruca daurica* (Joannis) belongs to Coleoptera, Chrysomelidae (Figure 1). It is reported to be mainly distributed in Mongolia, Russia (Siberia), North Korea, South Korea, and China, including Inner Mongolia, Xinjiang, and Gansu [34]. *G. daurica* has been a new pest in the grasslands of Inner Mongolia since its abrupt outbreak in 2009 [35]. This pest is an oligophagous herbivore, only feeding on *Allium* plants of the Liliaceae family, among which *A. mongolium* is the most suitable host plant [35]. Therefore, *G. daurica* must have the ability to detoxify or tolerate the main secondary metabolites in *Allium* plants. Our previous study showed that six metabolites in *A. mongolium*, including three flavonoids (isoquercitrin, isoflavone, and rutin) and three monosaccharides (β-d-glucopyranose, d-galactose, and l-rhamnose), affected the feeding preference of *G. daurica* [36]. For example, the addition of 0.1 mM isoflavones decreased the feed intake of larvae, and the feeding preference index decreased significantly. However, its mechanism of detoxification or tolerance remains unknown. In this study, these six metabolites in *A. mongolium* were respectively added to the diet for the third instar larvae of *G. daurica*. The purpose was to screen out the differentially expressed genes (DEGs) by performing high-throughput transcriptome sequencing and analyzing the detoxification metabolic pathways of this pest to host secondary metabolites so as to lay a foundation for further exploring the molecular mechanism of *G. daurica* adaptation to host plants.

## 2. Materials and Methods

### 2.1. Insects

The 1st and 2nd instar larvae were collected from the Siziwang grasslands of Inner Mongolia (42.2012° N, 112.0894° E), China, on 7 May 2023, and reared at 26 ± 1 °C, 60~80% relative humidity under a 16 h light/8 h dark period in a constant temperature incubator. The 3rd instar larvae were reared on *Allium tuberosum* for experiments.

### 2.2. Sample Processing

A total of 10% dimethyl sulfoxide (DMSO) solution was prepared with distilled water, which was used to prepare 0.1 mmol/L isoquercitrin (IQ), isoflavone (ISO), rutin (RT), d-galactose (Gal), β-d-glucopyranose (Glc), and l(+)-rhamnose (Rham) treatment solutions. Information on chemical substances and solvents is shown in Table S1.

A total of 7 experimental groups were set up, including 6 treatment groups and 1 solvent control group. Each insect box contained 20 3rd instar larvae of *G. daurica*. For the treatment groups, larvae fed on *A. tuberosum* soaked in 6 kinds of treatment solution for 5 min, respectively. For the solvent control group, larvae fed on the leaves of *A. tuberosum* soaked in 10% DMSO solution every day. Three days after feeding, 10 larvae in each insect box were collected for transcriptome sequencing. Three biological replicates were set up in each experimental group.

### 2.3. cDNA Library Construction and Transcriptome Sequencing

Transcriptome sequencing was performed by Ovison Gene Technology Co., Ltd. (Beijing, China). A total of 21 cDNA libraries were constructed, including 7 groups with 3 times per 10 larvae. RNA purity (OD260/280 and OD260/230) and RNA fragment length were measured by Nanodrop (Thermo Scientific, Thermo Fisher Scientific, Waltham, MA, USA) and Agilent 2100 (Agilent Technologies, Santa Clara, CA, USA) prior to library construction, and transcriptome sequencing was performed after qualification. The constructed libraries were sequenced using the Illumina Novaseq 6000 high-throughput sequencing platform, the raw data obtained by sequencing were filtered to remove the joint sequences and low-quality reads, and the high-quality sequences were finally obtained. Trinity v2.4.0 software [37] was used to splice and assemble clean read sequences to obtain unigenes.

### 2.4. Analysis and Annotation of Differentially Expressed Genes

DEseq1.10.1 software [38] was used to analyze the differentially expressed genes (DEGs), and the *p* value was corrected by the Benjamini–Hochbery (BH) method. The final corrected *p* value, i.e., the false discovery rate (*FDR*), was used as the key index for the screening of DEGs. Fold change (FC) represented the ratio of gene expression between samples of each treatment (IQ, ISO, RT, Gal, Glc, and RT) and control (DMSO). FDR < 0.05 and |log_2_(FC)| ≥ 1 were used as the screening criterion for DEGs. BLAST was used to compare the DEG sequences with Nr, Swiss-Port, GO, KOG, Pfam, and KEGG databases (*E* value < 1 × 10^−5^) to obtain the corresponding annotation information. GOseq v1.22.0 was used for enrichment analysis [39], the Cluster Profiler was used for KEGG pathway enrichment analysis, and the corrected *p* value (*q*) < 0.05 was used as the significantly enriched KEGG pathway.

### 2.5. Verification of Differentially Expressed Genes by qRT-PCR

Seven DEGs screened in Section 2.4 were randomly selected for qRT-PCR verification, including glutathione S transferase (GST, TRINITY_DN40461_c0_g1), pupal cuticle protein Edg-84A-like protein (CP, TRINITY_DN48195_c0_g1), elongation factor 1 alpha (ef1α, TRINITY_DN50797_c3_g2), cytochrome P450 insecticide resistance-associated cytochrome P450 (CYP450, TRINITY_DN47138_c1_g1), glycoside hydrolase family protein 48 (GH48, TRINITY_DN28963_c0_g1), chemosensory protein 2 (CSP2, TRINITY_DN44890_c0_g1), and peptidoglycan-recognition protein-SC2 (PGRPSC2, TRINITY_DN48140_c0_g2).

qRT-PCR was performed to confirm the reliability of the RNA sequencing results. The same samples were used as templates for sequencing. The primers were designed using the Primer Premier3.0 “https://bioinfo.ut.ee/primer3-0.4.0/ (accessed on 19 March 2024)” and synthesized by Sangon Biotech (Shanghai, China) (Table S2). qRT-PCR was conducted on the FTC-3000 (Funglyn Biotech, Toronto, Canada) using the MonAMP^TM^ SYBR^®^ Green qPCR Mix (Monad, Wuhan, China). The reaction procedure was as follows: 95 °C for 10 min followed by 40 cycles at 95 °C for 15 s and 60 °C for 1 min. The reaction procedure of the melting curve was 95 °C for 15 s, 60 °C for 15 s, and 95 °C for 15 s. Three technical replicates of each reaction were conducted, and *SDHA* was used as a reference gene [40]. The 2^−ΔΔCt^ method was used to calculate the relative expression levels [41].

## 3. Results

### 3.1. The Basic Characteristics of Transcriptome Sequencing Analysis

In order to clarify the effects of host plant metabolites on the gene expression in *G. daurica* larvae at the transcriptome level, twenty-one sequencing libraries were constructed from seven groups of the third instar larvae fed on the diet containing different metabolites. The raw data have been deposited in the National Center for Biotechnology Information (NCBI) under BioProject ID PRJNA1093491. A total of 472,847,958 raw read pairs were measured, and 469,646,761 clean read pairs were obtained after quality control. The clean data of each sample were 5.25~7.05 Gb, the G + C content was 34.56~37.13%, the error rate was 0.02~0.03%, the Q20 base ratio was 97.89~98.28%, and the Q30 base ratio was 93.78~94.76% (Table S3). A total of 124,348 unigenes were obtained, and N50 was 1127 bp. Among them, the number of sequences with a splicing length of 300 to 500 bp was 61,079, the number of sequences from 500 to 1000 bp was 36,605, the number of sequences from 1000 to 2000 bp was 16,919, the number of sequences greater than 2000 bp was 9745, and there were no sequences less than 300 bp (Figure S1). These results indicate that the transcriptome data are reliable and can be used for subsequent analysis.

### 3.2. Analysis and Annotation of Differentially Expressed Genes (DEGs)

**Figure 2:**
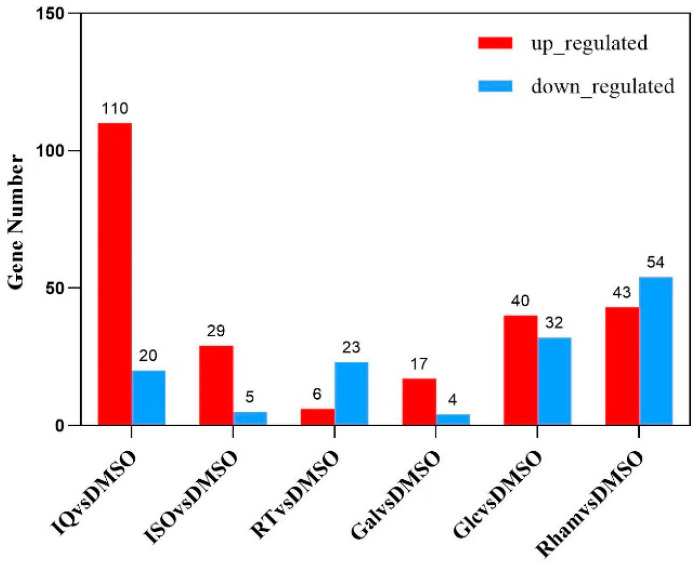
Statistical map of the gene number of the differentially expressed gene set. DMSO (control group): 10% dimethyl sulfoxide; IQ, ISO, RT, Gal, Glc, and RT (treatment groups): 0.1 mM isoquercitrin, isoflavone, rutin, d-galactose, β-d-glucopyranose, and l(+)-rhamnose, respectively. Three replicates were set up in each experimental group, and each repeat included 10 larvae.

**Figure 3:**
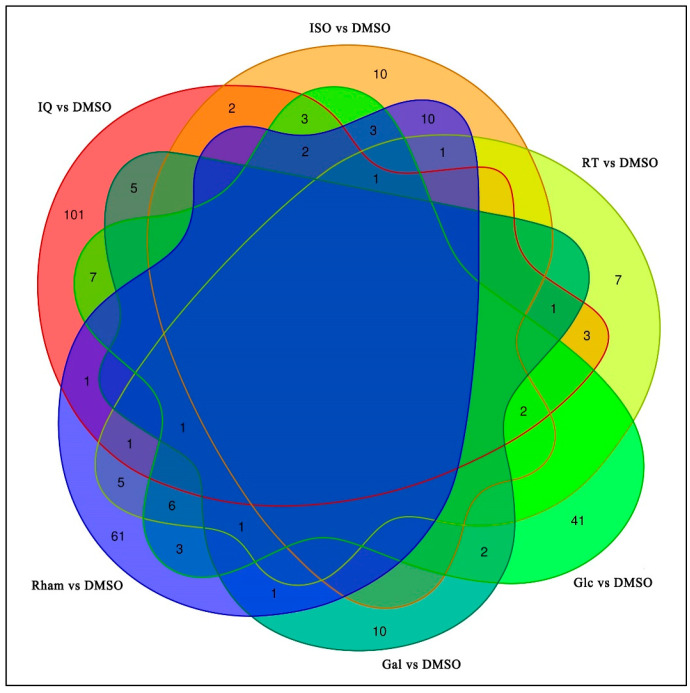
Venn diagram of differentially expressed genes. Red outline area: IQ vs. DMSO; orange outline area: ISO vs. DMSO; yellow outline area: RT vs. DMSO; green outline area: Glc vs. DMSO; cyan outline area: Gal vs. DMSO; blue outline area: Rham vs. DMSO.

In order to screen out the DEGs between different treatments and the control, the expression data were statistically analyzed. The results showed that compared with the control, a total of 291 DEGs were detected after larvae were fed on the diet containing six different metabolites, respectively, and the treatment groups of isoquercitrin, isoflavone, rutin, d-galactose, β-d-glucopyranose, and l-rhamnose had 110, 29, 6, 17, 40, and 43 up-regulated DEGs and 20, 5, 23, 4, 32, and 54 down-regulated DEGs, respectively (Figure 2). In order to better reflect the effects of feeding different substances on the transcriptional level in larvae, a Venn diagram was drawn with *FPKM* > 1 as the criterion for judging gene expression (Figure 3). In primary metabolite treatment groups, the l-rhamnose treatment group contained the highest number of DEGs (97), and the d-galactose treatment group contained the lowest (21). In secondary metabolite treatment groups, the isoquercitrin treatment group contained the highest number of DEGs (130), and the rutin treatment group contained the lowest (29).

The DEGs are listed in Table S4. Among them, there were twenty-six major DEGs (FDR < 0.01 with functional annotations) in the isoquercitrin treatment group (twenty-two up-regulated and four down-regulated). The up-regulated genes mainly included dehydrogenase, glycoside hydrolase family protein 48, cytochrome P450, cytochrome b, glycoside hydrolase family 1, transposase, and solute carrier family 13 member 3. The down-regulated genes included elongation factor 1-alpha, C-type lectin precursor, coat protein, and cytochrome c oxidase subunit I. There were fifteen major DEGs in the isoflavone treatment group (fourteen up-regulated and one down-regulated). The up-regulated genes mainly included ribosomal proteins, solute carrier family 13 member 3, glyceraldehyde-3-phosphate dehydrogenase, cytochrome b, and cytochrome c oxidase subunit II. The down-regulated gene was cytochrome c oxidase subunit I. There were seven major DEGs in the rutin treatment group (three up-regulated and four down-regulated). The up-regulated gene was Ferritin, and the down-regulated genes included cytochrome c oxidase subunit I, peptidoglycan-recognition protein-SC2, polyprotein, and 31 kDa antigen. In the β-d-glucopyranose treatment group, there were 24 major DEGs (10 up-regulated and 14 down-regulated). The up-regulated genes mainly included Chlorophyll a-b-binding protein 40, reverse transcriptase homolog, and solute carrier family 13 member 3. The down-regulated genes mainly included 31 kDa antigens, hemoglobin subunit beta, peptidoglycan-recognition protein-SC2, cytochrome c oxidase subunit I, elongation factor 1-alpha 1-like protein, Kruppel-like protein 1, and Ferritins. In the l-rhamnose treatment group, there were 40 major DEGs (25 up-regulated and 15 down-regulated). The up-regulated genes mainly included cytochrome c oxidases subunit I, heat shock proteins, glyceraldehyde-3-phosphate dehydrogenase, and ribosomal proteins. The down-regulated genes mainly included 31 kDa antigen, hemoglobin subunit beta, attacin-like immune proteins, peptidoglycan-recognition protein-SC2, and defensin 1 precursor. In the d-galactose treatment group, there was only one up-regulated major gene (phosphoserine aminotransferase).

### 3.3. GO and KEGG Enrichment Analysis of Differentially Expressed Genes (DEGs)

**Figure 4:**
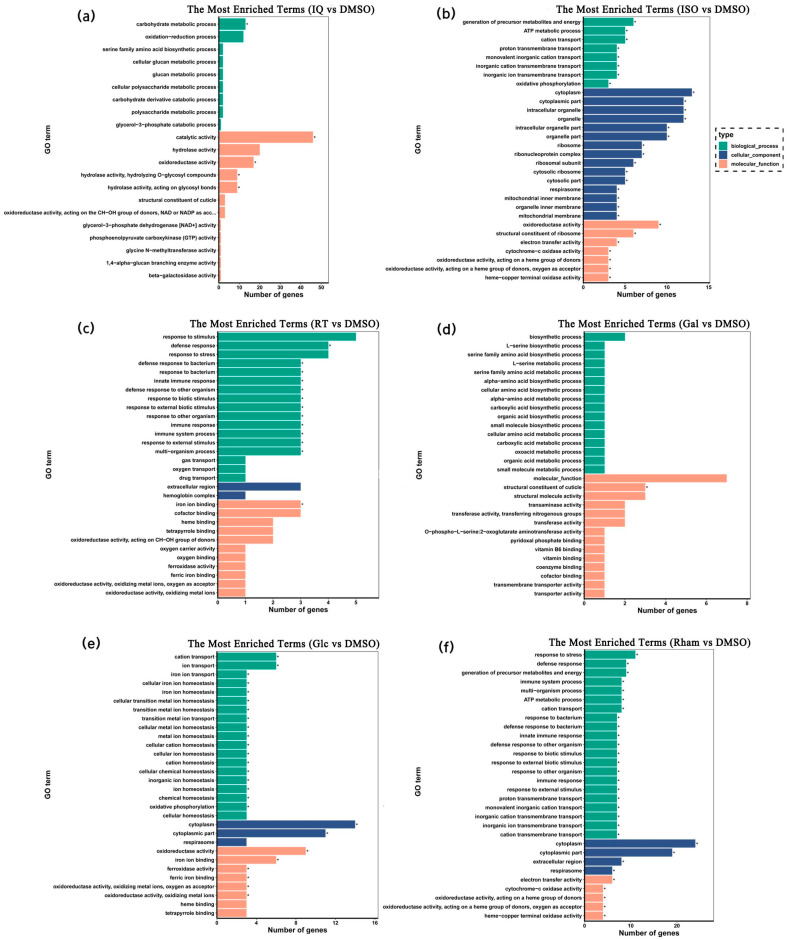
GO enrichment analysis of differentially expressed genes in the larva of *G. daurica*. (**a**–**f**) indicate isoquercitrin, isoflavone, rutin, d-galactose, β-d-glucopyranose, and l(+)-rhamnose 6 different treatments, respectively. *: significant enrichment with *p* value (*q*) < 0.05.

To further understand the function of DEGs, these DEGs were subjected to GO and KEGG enrichment analysis. GO enrichment showed that most GO terms were enriched in biological processes and molecular functions, and there were fewer in cellular components (Figure 4). In the isoquercitrin treatment, most DEGs were significantly enriched in catalytic activity, and, secondly, oxidoreductase activity and carbohydrate metabolic process. In the isoflavone treatment, most DEGs were significantly enriched in cellular components. For molecular functions, most DEGs were significantly enriched in oxidoreductase activity, and, secondly, structural constituent of ribosome. For biological processes, most DEGs were significantly enriched in the generation of precursor metabolites and energy, and, secondly, ATP metabolic process and cation transport. In the rutin treatment, most DEGs were significantly enriched in GO terms related to defense and immune responses. In the d-galactose treatment, the structural constituent of the cuticle was only one significantly enriched GO term. In the β-d-glucopyranose treatment, most DEGs were significantly enriched in the cytoplasm, and, secondly, the cytoplasmic part. For molecular functions, oxidoreductase activity was the GO term significantly enriched by most DEGs, and secondly, iron ion binding. For biological processes, nearly all DEGs were significantly enriched in GO terms related to ion transport and homeostasis. In the l-rhamnose treatment, most DEGs were also significantly enriched in the cytoplasm, and, secondly, the cytoplasmic part. For biological processes, nearly all DEGs were significantly enriched in GO terms related to various responses and transmembrane transports. For molecular functions, most DEGs were significantly enriched in electron transfer activity.

**Figure 5:**
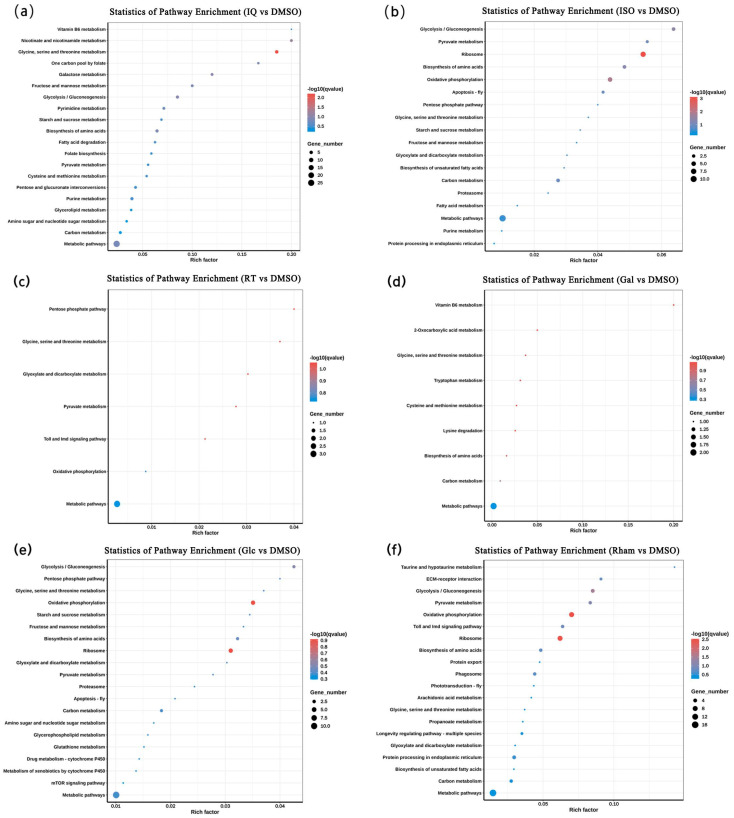
KEGG enrichment analysis of differentially expressed genes in the larva of *G. daurica*. (**a**–**f**) indicate isoquercitrin, isoflavone, rutin, d-galactose, β-d-glucopyranose, and l(+)-rhamnose 6 different treatments, respectively.

KEGG enrichment analysis showed that most DEGs were enriched in various metabolism pathways (Figure 5). For all six metabolites, glycine, serine, and threonine metabolism was only one commonly enriched KEGG pathway. Common enriched KEGG pathways were carbon metabolism and pyruvate metabolism for three primary metabolites and three secondary metabolites, respectively. In the isoquercitrin treatment, the pathways with the highest enrichment intensity included vitamin B6 metabolism, nicotinate and nicotinamide metabolism, glycine, serine, and threonine metabolism, and one carbon pool by folate. In the isoflavone treatment, the pathways with the highest enrichment intensity included glycolysis/gluconeogenesis, pyruvate metabolism, ribosome, and biosynthesis of amino acids. In the rutin and d-galactose treatments, fewer DEGs were enriched in fewer KEGG pathways, and the pathways with the highest enrichment intensity were the pentose phosphate pathway in the rutin treatment and vitamin V6 metabolism in the d-galactose treatment. In the β-d-glucopyranose treatment, the pathways with the highest enrichment intensity included glycolysis/gluconeogenesis and the pentose phosphate pathway. In the l-rhamnose treatment, the pathways with the highest enrichment intensity included taurine and hypotaurine metabolism, ECM–receptor interaction, glycolysis/gluconeogenesis, and pyruvate metabolism.

### 3.4. Validation of Differentially Expressed Genes (DEGs)

**Figure 6:**
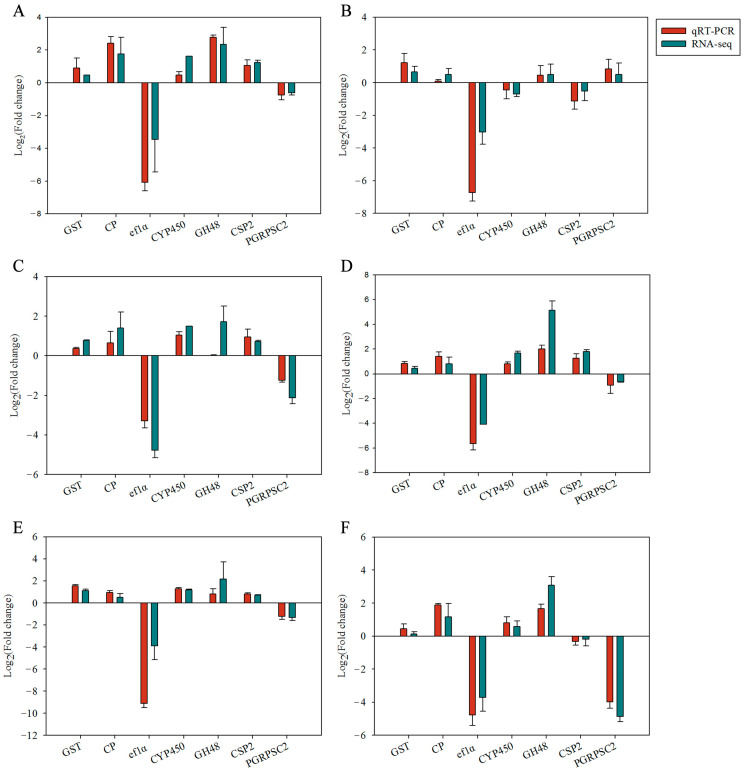
Validation of RNA-seq-derived changes in transcript abundance with qRT-PCR for 7 differentially expressed genes. Fold change: normalization values RNA sequencing data and relative expression values obtained with qRT-PCR based on the 2^−ΔΔCt^ method. (**A**) Isoquercitrin; (**B**) isoflavone; (**C**) rutin; (**D**) d-galactose; (**E**) β-d-glucose; (**F**) l-rhamnose.

According to the results of transcriptome DEG analysis, seven DEGs (GST, CP, ef1α, CYP450, GH48, CSP2, and PGRPSC2) were randomly selected for qRT-PCR verification (Figure 6). The results showed that qRT-PCR and RNA-seq trends of seven DEGs were completely consistent, indicating that the transcriptome sequencing results of this experiment are reliable.

## 4. Discussion

Herbivorous insects have a very close relationship with host plants, which constitute the food source, mating site, oviposition site, and habitat of all or part of their life cycle, and the digestion and utilization of nutrients (plant primary metabolites) and the adaptation of secondary metabolites are the key factors for insects to establish populations [42]. In this study, after the larvae of *G. daurica* fed, respectively, on foods containing six different plant metabolites (three primary metabolites and three secondary metabolites), most DEGs were enriched in various metabolic pathways, suggesting that host plants may affect the biology of herbivorous insects by mainly mediating their various metabolic processes. Glycine, serine, and threonine metabolism was the only common enriched KEGG pathway by the DEGs among the six metabolite treatments, indicating that both primary and secondary metabolites can affect amino acid metabolism. Carbon metabolism was the only common enriched KEGG pathway by the DEGs among the three primary metabolites (carbohydrates), showing that carbohydrates in host plants mainly affect the absorption, transportation, decomposition, and synthesis of carbon in herbivorous insects. Plant secondary metabolites can affect insect feeding, growth and development, and reproduction, and even kill insects with toxicity [43]. The adaptation process of insects to plant secondary metabolites requires the participation of a variety of enzymes, and feeding on foods containing different secondary metabolites will affect the expression of genes. Six CYP450 genes and two esterase genes were up-regulated in the isoquercitrin treatment for this paper, which is similar to the results of others. For example, in *Spodoptera frugiperda*, CYP450 activity was induced to increase by a variety of secondary metabolites, such as flavonoids, menthol, or indole-3-carbinol, and was also involved in the metabolism of multiple secondary metabolites such as indole, coumarin, flavonoids, and glucosinoside [30]. Ferulic acid induced the expression of GST family genes in *Sitodiplosis mosellena* [44].

Cytochrome b is a component of the mitochondrial respiratory chain, is involved in electron transport in the respiratory chain, and is closely related to cellular energy metabolism [45]. Cytochrome b is oxidized by the cytochrome c oxidase, which finally converts molecular oxygen (O_2_) to water (H_2_O) [46]. In the present study, multiple genes encoding cytochrome b and cytochrome c oxidase (COI and COII) were differentially expressed in the other treatment groups, except for d-galactose, especially two COI, two COII, and two cytochrome b in the l(+)-rhamnose treatment and two COI, one COII, and one cytochrome b in the isoflavone treatment, which suggests that these plant metabolites may have an important effect on energy metabolism in *G. daurica*.

l-rhamnose is a monosaccharide that is widely present in nature, especially in some plants or microbial polysaccharides with anti-tumor activity, and it has functions such as regulating carbohydrate and lipid metabolism in animals [47,48,49]. In the l-rhamnose treatment, we found that the down-regulated genes mainly included 31 kDa antigen, attacin-like immune proteins, peptidoglycan-recognition protein-SC2, and defensin 1 precursor, which suggests that l-rhamnose may be related to the immune defense in *G. daurica*.

Ribosome is a kind of ribonucleoprotein particle in biological cells whose main function is to synthesize amino acids into protein polypeptide chains with mRNAs as the guide, and it is an important organelle for protein synthesis in cells [50]. In this study, a large number of ribosomal protein genes were up-regulated in the isoflavone and l-rhamnose treatments, which implies that isoflavone and l-rhamnose may have significant effects on protein synthesis in *G. daurica*.

In summary, the addition of six different substances to the diet mainly affected the ribosome and metabolic pathways of *G. daurica*, and the adaptation of larvae to *allium* host may be through the synthesis of corresponding proteins and the acceleration of metabolism.

## 5. Conclusions

A total of 291 DEGs were identified compared to the solvent control (DMSO). The main DEGs in the isoquercitrin, isoflavone, rutin, d-galactose, β-d-glucopyranose, and l-rhamnose treatment groups were 130, 34, 29, 21, 72, and 97, respectively. GO and KEGG enrichment analysis showed that most DEGs were enriched in various metabolic pathways, implying that these six main primary and secondary metabolites in *Allium* plants may affect various metabolic processes in the larvae of *G. daurica*.

## Data Availability

Data are contained in the article and Supplementary Materials. The raw data have been deposited in the National Center for Biotechnology Information (NCBI) under BioProject ID PRJNA1093491.

## Supplementary Materials

The following supporting information can be downloaded at: https://www.mdpi.com/article/10.3390/insects15110847/s1, Figure S1: Length distribution of unigenes and transcripts; Table S1: List of Chemical and solvent information; Table S2: List of primers of qRT-PCR; Table S3: Statistical table of sequencing data; Table S4: Differentially expressed genes.
