# Supplementary material for: Comparative Transcriptome Analysis of the Pest Galeruca daurica (Coleoptera: Chrysomelidae) Larvae in Response to Six Main Metabolites from Allium mongolicum (Liliaceae)
Source: Insects. 2024 Oct 29;15(11):847. doi: 10.3390/insects15110847 (PMC11594626; doi:10.3390/insects15110847)
Supplement: Supplementary file 1 [file insects-15-00847-s001.zip › Table S3 Statistical table of sequencing data.pdf]

Table S3 Statistical table of sequencing data

Sample name	Raw reads	Raw bases	Clean reads	Clean bases	Error rate	Q20	Q30	GC content
IQ_1	37762394	5.67G	37522202	5.67G	0.03%	98.08%	94.04%	35.57%
IQ_2	39040988	5.87G	38852494	5.87G	0.03%	98.04%	94.02%	36.82%
IQ_3	34899332	5.25G	34679098	5.24G	0.03%	97.90%	93.78%	35.65%
ISO_1	40206856	6.03G	39889606	6.02G	0.02%	98.18%	94.41%	37.13%
ISO_2	46908650	7.05G	46262464	6.99G	0.02%	98.03%	94.76%	36.43%
ISO_3	38240394	5.75G	37998342	5.74G	0.03%	97.95%	94.02%	35.99%
RT_1	38912168	5.86G	38717084	5.85G	0.03%	97.97%	93.91%	35.60%
RT_2	36591854	5.51G	36430798	5.5G	0.03%	97.98%	93.84%	36.13%
RT_3	40319392	6.06G	40097582	6.05G	0.03%	97.93%	93.90%	35.84%
Gal_1	40296876	6.06G	40102698	6.06G	0.02%	98.28%	94.64%	36.82%
Gal_2	38630970	5.8G	38373902	5.79G	0.03%	97.97%	93.91%	35.67%
Gal_3	44671300	6.72G	44440026	6.71G	0.03%	98.10%	94.26%	36.02%
Glc_1	41653336	6.27G	41452756	6.26G	0.03%	98.07%	94.10%	36.25%
Glc_2	39553034	5.93G	39220522	5.92G	0.03%	98.05%	94.03%	34.68%
Glc_3	35857230	5.36G	35460672	5.35G	0.02%	98.27%	94.65%	36.43%
Rham_1	38872526	5.85G	38678066	5.84G	0.03%	98.04%	94.03%	35.63%
Rham_2	38441528	5.78G	38217226	5.77G	0.03%	98.05%	94.01%	35.69%
Rham_3	38284406	5.76G	38097704	5.75G	0.03%	98.04%	93.99%	34.56%
DMSO_1	38013462	5.71G	37746128	5.7G	0.03%	98.02%	94.01%	35.57%
DMSO_2	38097304	5.73G	37867212	5.72G	0.03%	97.89%	93.79%	36.74%
DMSO_3	38653974	5.78G	38211284	5.77G	0.03%	97.98%	93.97%	35.58%
