# Supplementary material for: Comparative Transcriptome Analysis of the Pest Galeruca daurica (Coleoptera: Chrysomelidae) Larvae in Response to Six Main Metabolites from Allium mongolicum (Liliaceae)
Source: Insects. 2024 Oct 29;15(11):847. doi: 10.3390/insects15110847 (PMC11594626; doi:10.3390/insects15110847)
Supplement: Supplementary file 1 [file insects-15-00847-s001.zip › Table S1 List of Chemical and solvent information.pdf]

Table S1 List of Chemical and solvent information

Chemical substance name	Molecular formula	Molecular weight	CAS number	Purity (%)
Isoquercitrin, IQ	$C_{21}H_{20}O_{12}$	464.38	482-35-9	98
Isoflavone, ISO	$C_{15}H_{10}O_2$	222.24	574-12-9	BR, ≥40
Rutin, RT	$C_{27}H_{30}O_{16}$	610.52	153-18-4	BR,95
D-Galactose, Gal	$C_6H_{12}O_6$	180.16	59-23-4	99
β-D-Glucopyranose, Glc	$C_6H_{12}O_6$	180.16	492-61-5	85
L(+)-Rhamnose, Rham	$C_6H_{14}O_6$	182.17	10030-85-0	99
Dimethyl sulfoxide, DMSO	C_2H_6OS	78.13	67-68-5	99.9
