# Supplementary material for: Comparative Transcriptome Analysis of the Pest Galeruca daurica (Coleoptera: Chrysomelidae) Larvae in Response to Six Main Metabolites from Allium mongolicum (Liliaceae)
Source: Insects. 2024 Oct 29;15(11):847. doi: 10.3390/insects15110847 (PMC11594626; doi:10.3390/insects15110847)
Supplement: Supplementary file 1 [file insects-15-00847-s001.zip › Table S2 List of primers of qRT-PCR.pdf]

Table S2 List of primers of qRT-PCR

Gene names	Forward primer（5’ to 3’）	Reverse primer（5’ to 3’）
SDHA	GGGAGACCACAATCTCCTCA	AGCTGGTGCTCCTAAGTCCA
GST	GATGTTGGGTCCTCCCACTA	TGTAGCCATCATCGTCCAAA
CP	CCCTCATACCGGAGATGCTA	AAGTAACTGGGGCCGCTAAT
ef1α	AGGTCATCGTCCTCAACCAC	CTTGGAGGGAACCATCTTGA
CYP450	TTGGGCCAACTTGTTGTGTA	AAATGCCGTTCACGATTTTC
GH48	TGTCAACACCTTCCACGGTA	CCTCCCAATCCAAAGTGCTA
CSP2	CCCAAAAACCTTCGAAGAAA	CGTCGCATCCTTCACAATTT
PGRPSC2	GCAAGTCCTCCGAATGATGT	GAACACAGTGAGCTCCACGA
