# Supplementary figures and images for: Comparative Transcriptome Analysis of the Pest Galeruca daurica (Coleoptera: Chrysomelidae) Larvae in Response to Six Main Metabolites from Allium mongolicum (Liliaceae)
Source: Insects. 2024 Oct 29;15(11):847. doi: 10.3390/insects15110847 (PMC11594626; doi:10.3390/insects15110847)

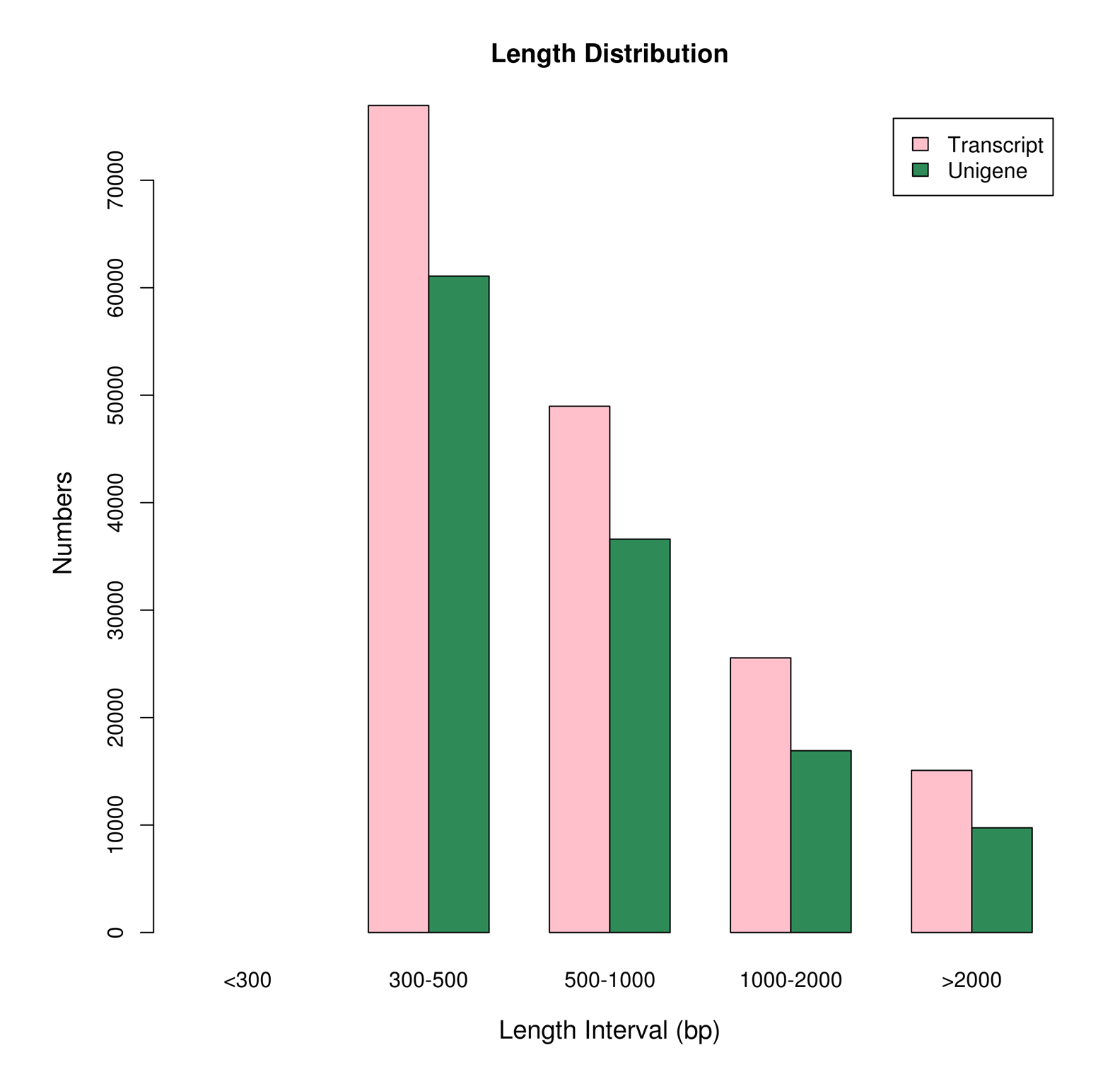

Supplement: Supplementary file 1 [file insects-15-00847-s001.zip › Fig.S1.jpg]
